# Wellbeing and mental health in Chilean public-school students: emotion regulation and character strengths

**DOI:** 10.3389/fpsyg.2026.1845218

**Published:** 2026-08-06

**Authors:** Paulina Ortiz-Correa, María Josefina Escobar, Carolina Panesso Giraldo, Valeria Salinas, Regina Rivera

**Affiliations:** 1 School of Psychology, Universidad Adolfo Ibáñez, Peñalolén, Chile; 2 Center for Social and Cognitive Neuroscience, Universidad Adolfo Ibáñez, Las Condes, Chile; 3 Parenting, Care and Childhood Unit (ANIDA), Universidad Adolfo Ibáñez, Santiago, Chile; 4 Corporación Municipal de Peñalolén, Educación REDUCA, Santiago, Chile

**Keywords:** emotion regulation, character strengths, wellbeing, children, adolescents, schools, public education, positive education

## Abstract

**Introduction:**

This study examined the associations between emotion regulation, character strengths, and wellbeing among Chilean students attending public schools in Peñalolén, Santiago.

**Methods:**

Participants were 3,569 children and adolescents aged 8–18 years who completed the EPOCH Well-being Scale, selected item-level indicators of the CERQ-K (younger students) or DERS-E (older students), and 15 items from the VIA Character Strengths Inventory. Five hierarchical regression models were estimated for each EPOCH domain, separately by age group.

**Results:**

Adaptive emotion regulation strategies predicted higher happiness, optimism, and perseverance among younger students, whereas emotional difficulties predicted lower wellbeing among older students. Adding character strengths significantly increased explained variance, particularly for perseverance and optimism. Love of learning, hope, self-regulation, enthusiasm, love, and teamwork consistently predicted wellbeing.

**Discussion:**

Findings support the integration of emotion regulation and strengths-based education to promote student wellbeing in Latin American school settings.

## 1 Introduction

Mental health and wellbeing have taken on an increasingly central role following the impact of the COVID-19 pandemic on school communities worldwide. Rates of depression and anxiety among children and adolescents rose markedly during this period (Marques de Miranda et al., 2020), compounding pre-existing vulnerabilities in Latin America, where many young people already faced mental health challenges without adequate support. In Chile, 44% of adolescents reported psychological problems before the pandemic, with a sharp increase in anxiety, stress, and depressive symptoms afterward (Universidad de Chile, 2021). National data confirm this burden: the 10th National Youth Survey found that one in four young people (26.9%) experience moderate-to-severe depressive or anxious symptoms, twice the adult rate, with marked gender and socioeconomic disparities (Instituto Nacional de la Juventud, 2022), and the First National Report on the Wellbeing of Children in Chile documented a rise in depressive symptoms from 14% in 2017 to 23% in 2022, alongside inequities by school type, highlighting the particular vulnerability of public-school students (Observatorio Niñez, 2024).

Students in public schools serving low-income communities face cumulative risks, including economic stress, exposure to violence, and academic under-resourcing, while access to psychosocial support remains limited, and the pandemic further disrupted school routines and family stability (Escobar et al., 2021; Martínez-Líbano and Yeomans-Cabrera, 2024). This concentration of risk in public education makes it a priority setting for wellbeing research and intervention Positive psychology reoriented the study of mental health from deficit and disorder toward the psychological resources that allow young people to function well and experience their lives as meaningful (Seligman and Csikszentmihalyi, 2000). In educational settings, this reorientation is best captured by multidimensional accounts of student wellbeing. In response, international organizations such as UNESCO (2024) have emphasized integrating social and emotional learning (SEL) and wellbeing promotion into education systems. Within this movement, Positive Education, which merges positive psychology and pedagogy, has emerged as a promising framework to foster resilience, strengths, and flourishing among students (Seligman et al., 2009; Waters and Loton, 2019). The EPOCH model, comprising Engagement, Perseverance, Optimism, Connectedness, and Happiness, adapts Seligman’s (2011) adult framework to adolescence and treats wellbeing not as a single index but as a profile of distinct, cultivable dimensions, each potentially supported by different underlying resources (Kern et al., 2016). Evidence indicates that these dimensions are responsive to change: school-based positive psychology interventions produce small but reliable gains in adolescents’ wellbeing and reductions in distress, both immediately and when wellbeing is assessed over longer follow-up periods (Tejada-Gallardo et al., 2020; Vella-Brodrick et al., 2025). These benefits extend across diverse cultural settings, including large-scale programs in Bhutan, Mexico, and Peru (Adler, 2016), and recent randomized trials confirm improvements in wellbeing and resilience among school students (Zhang et al., 2025). Yet in Latin America, and particularly in Chile, this evidence base remains thin (Ministerio de Educación, 2022, 2023), and little is known about which specific resources underlie each dimension of wellbeing in school-aged populations.

Two psychological resources have emerged as especially relevant to wellbeing within this framework: emotion regulation and character strengths. Emotion regulation refers to the processes by which individuals influence which emotions they have, when they have them, and how they experience and express them (Gross, 2015). It is particularly consequential during childhood and adolescence, a developmental window marked by rapid socio-emotional change and by still-maturing neural systems that support regulatory control (Silvers, 2022). Difficulties in emotion regulation prospectively predict internalizing problems across development (Murray et al., 2025), whereas adaptive strategies such as cognitive reappraisal and perspective taking buffer against distress and support psychological adjustment (Caqueo-Urízar et al., 2020; Martínez-Líbano et al., 2025). Importantly, the regulatory repertoire shifts with age. Younger children draw on emerging cognitive strategies, whereas adolescents contend with more intense affective experiences, so that the relative absence of regulatory difficulties becomes increasingly central to their wellbeing (Gross, 2015; Silvers, 2022).

Character strengths offer a second, dispositional route to wellbeing. Within the Values in Action framework, they are understood as a family of positively valued characteristics that people recognize in themselves and enact across situations (Peterson and Seligman, 2004). In children and adolescents, strengths such as hope, zest, gratitude, and love of learning show consistent positive associations with life satisfaction and school engagement, and inverse associations with depression and anxiety (Park and Peterson, 2006; Oguni et al., 2025). Importantly, a recent meta-analysis of more than one hundred studies found that hope and zest are the strongest correlates of wellbeing, and that strength–wellbeing associations are consistently larger than strength–psychopathology ones, positioning strengths as distinct pathways to positive functioning rather than the mere inverse of distress (Casali and Feraco, 2025).

Although emotion regulation and character strengths have each been robustly linked to wellbeing, they are rarely examined together, and their joint contribution remains poorly understood. Conceptually, the two are complementary rather than redundant: emotion regulation concerns how affective experience is managed as it unfolds, whereas character strengths reflect the motivational and dispositional resources that give behavior direction and meaning (Niemiec, 2023; Peterson and Seligman, 2004). Because they capture partly distinct aspects of positive functioning, strengths may relate to wellbeing in ways not fully accounted for by regulatory capacities. Although the wellbeing benefits of character strengths are well documented, far less is known about how they operate alongside emotion regulation. Whether strengths account for variance in wellbeing beyond that explained by regulatory capacities, and which particular strengths matter for which wellbeing dimensions across developmental stages, remain open questions in school-aged populations.

This gap is especially pronounced in Latin America and virtually unaddressed in Chile, where national assessments still focus predominantly on risk and deficit indicators, leaving protective assets such as strengths and wellbeing largely unexplored. The Chilean validation of the EPOCH Measure of Adolescent Wellbeing, which captures five positive dimensions (Engagement, Perseverance, Optimism, Connectedness, and Happiness) and represents a developmentally appropriate translation of the adult PERMA framework (Kern et al., 2016; Ortiz-Correa et al., 2020), offers a validated tool for advancing this agenda. Yet no large-scale Chilean study has examined how emotion regulation and character strengths jointly predict the multiple dimensions of wellbeing in school-aged populations.

Within this context, the present study sought to examine the relationships between emotion regulation, character strengths, and wellbeing among children and adolescents attending public schools in Peñalolén, Chile. By integrating selected item-level indicators derived from the EPOCH for multidimensional wellbeing, CERQ-K for cognitive emotion regulation (children), and DERS-E for emotion-regulation difficulties (adolescents), alongside a measure of character strengths, the study provides novel empirical evidence from Latin America on how psychological resources interact to shape youth wellbeing. To our knowledge, this is the first study in Latin America to simultaneously examine these variables in school-aged populations.

This study aimed to examine whether selected item-level indicators of emotion regulation and character strengths predict the five dimensions of wellbeing (Engagement, Perseverance, Optimism, Connectedness, Happiness) in children and adolescents. We hypothesized that selected indicators of adaptive regulation strategies and specific character strengths, particularly *Hope*, *Love of Learning*, and *Teamwork*, would be associated with higher EPOCH scores.

## 2 Hypotheses

Based on developmental and positive psychology frameworks, we hypothesized that selected indicators of emotion regulation and character strengths would be differentially associated with wellbeing across age groups. Specifically, we expected that adaptive emotion regulation strategies would be positively associated with happiness, optimism, and perseverance among younger students, whereas greater emotion regulation difficulties would be negatively associated with wellbeing among older adolescents. We further hypothesized that the inclusion of the selected item-level indicators of character strengths would significantly increase the explained variance in all wellbeing domains beyond emotion regulation, particularly for perseverance and optimism. Finally, we expected that strengths reflecting cognitive–transcendence (e.g., love of learning, hope, self-regulation) and affective–interpersonal orientation (e.g., love, teamwork, enthusiasm) would emerge as consistent predictors of wellbeing across age groups.

## 3 Method

### 3.1 Research design

A quantitative cross-sectional study was conducted to examine emotional regulation strategies, trusted adult figures within the school setting, and the strengths of students from the 15 schools managed by the Municipal Corporation of Peñalolén (CORMUP). To assess these aspects, a survey was conducted among students. For the Special Education School, a separate version of the survey was administered and completed by students’ caregivers. The survey comprised both questions specifically designed for this study and items selected from the EPOCH Well-being Scale and the VIA Inventory of Strengths.

### 3.2 Participants

Participants were students from 3rd grade to 11th grade from all 15 municipal schools in the commune of Peñalolén. A total of 3,569 students participated in the study. Regarding gender, 45.6% identified as female, 52.1% as male, and the remaining 2.3% preferred not to specify their gender. Among students aged 8–12 years (*n* = 2.523), 51% identified as male, 47% as female, and 2% as another or unspecified gender. Among those aged 13 years and older (n = 1.046), 53% identified as male, 44% as female, and 3% as another or unspecified gender. The distribution of participants by age group is shown in Table 1, with a *younger group* (8–12 years) and an *older group* (13–18 years), following the age categories of the instruments used.

**Table 1:** Distribution of participants by age group.

Age group	Number of students	Percentage (%)
Younger group (8 to 12 years old)	2,523	70%
Older group (13 to 18 years old)	1,046	30%
Total	3,569	100%

Previous research has shown that municipal public schools in Chile serve a higher proportion of socioeconomically disadvantaged students than private voucher schools (Elacqua, 2009). Although individual socioeconomic characteristics were not collected in the present study, describing the educational setting provides important context for interpreting the findings.

### 3.3 Data collection tools

Data were collected in 2024 using anonymous digital surveys approved by the UAI Ethics Committee. Before beginning the survey, all participants completed an informed assent that explained the voluntary nature of their participation, assured the confidentiality of their responses, and emphasized their right to withdraw from the study at any time. The survey included:

#### 3.3.1 Sociodemographic questionnaire

Covering age, educational level, and gender, as well as questions about the presence of trusted adults they could turn to when feeling worried or sad, particularly within the school environment.

#### 3.3.2 Item-selection rationale

Because data collection was embedded in a large-scale school-based assessment, the participating schools provided only a limited administration window during regular class time. Therefore, administering the complete versions of all instruments was not feasible. To reduce participant burden while preserving coverage of the main theoretical domains, we used selected items derived from previously validated instruments rather than the complete scales. This was not a validation of short forms; it was an item-level indicator approach informed by prior psychometric evidence. Items were selected based on four criteria: (a) theoretical relevance to the target domain, (b) developmental appropriateness and clarity for school-aged respondents, (c) suitability for the school context, and (d) when available, evidence from prior validation studies or confirmatory factor analyses showing strong factor loadings on the intended construct. In CFA terms, items with higher loadings are stronger observed indicators of their latent factor. Nevertheless, the resulting variables should be interpreted as single-item indicators of the corresponding domains, not as psychometrically equivalent to the full validated scales.

EPOCH well-being scale, which is based on the PERMA model. This self-report questionnaire measures psychological functioning and mental health in young people. It consists of 20 items grouped into five dimensions of positive psychological traits: Engagement, Perseverance, Optimism, Connectedness, and Happiness. Each item is rated on a 5-point Likert scale, ranging from “almost never” to “almost always.” Scores are calculated separately for each dimension (Ortiz-Correa et al., 2020). Confirmatory factor analyses supported the five-factor structure, with each dimension showing good internal consistency, as well as convergent and divergent validity. These results support the use of the EPOCH scale as a reliable and valid instrument for assessing positive functioning in adolescents in the Chilean context (Ortiz-Correa et al., 2020). The original EPOCH Measure of Adolescent Wellbeing was developed by Kern et al. (2016) to assess five positive psychological dimensions—Engagement, Perseverance, Optimism, Connectedness, and Happiness in adolescents, providing a multidimensional framework for understanding wellbeing during this developmental stage.

The selected items from this scale, along with the psychological traits they represent, are described below. These items were used to assess key dimensions of adolescent wellbeing. The item “When I learn something new, I lose track of time” reflects the dimension of engagement. “I am very studious and hardworking” corresponds to Perseverance. “In moments of doubt and uncertainty, I expect the best” relates to optimism, and “I have friends I care about deeply” represents connectedness. These items were selected for their relevance to the EPOCH model and their suitability for assessing positive psychological functioning in school-aged youth.

The VIA character strengths scale, is a self-report instrument consisting of 24 items, each rated on a five-point Likert scale ranging from “very much like me” to “not at all like me.” Each item assesses one of the 24-character strengths included in the VIA classification developed by Peterson and Seligman (2004). For this study, we selected 15 items from the original scale, which are listed below: *I know my strengths; I recognize the strengths of my classmates; I seek new opportunities to learn; I help and care for others; Even when I’m afraid, I show courage; I go through life feeling cheerful; I enjoy making friends; I can put myself in others’ shoes—I am empathetic; I like to participate and collaborate in groups; I treat others the way I would like to be treated; I avoid doing things I might regret later; I can manage my impulses and emotions; I’m optimistic and tend to expect the best; I give thanks for all the good things that happened to me; I try to overcome every obstacle I encounter*. These items were selected to represent a diverse set of character strengths relevant to the school context and psychological wellbeing.

The cognitive emotion regulation questionnaire – kids (CERQ-K) is an instrument developed to examine the cognitive strategies that children and adolescents use after experiencing a negative event. The CERQ-K consists of 36 items grouped into five factors: Self-blame, Rumination, Proactive Thinking, Distraction, and Perspective. These factors have demonstrated acceptable internal consistency, with Cronbach’s alpha coefficients ranging from 0.65 to 0.80. For this study, we selected the item with the highest loading from each factor. The scale has been validated for Latin American child and adolescent populations (Lemos et al., 2021). The selected items from the CERQ-K scale, administered to students aged 8 to 12, reflect five key cognitive emotion regulation strategies. These include: “I keep thinking about how horrible what happened was” (Rumination), “I think it all happened because of me” (Self-blame), “I think about how I can change what happened to me” (proactive thinking), “I think about something nice instead of what happened” (Distraction), and “I think it’s not as bad as other things that could happen” (perspective). These items were chosen to represent both maladaptive and adaptive regulation processes.

The difficulties in emotion regulation scale (DERS-E) was used to assess problems related to emotional regulation. This scale is based on an integrative conceptualization of emotion regulation that encompasses not only the modulation of emotional arousal, but also the awareness, understanding, and acceptance of emotions, as well as the ability to act in a desired way regardless of emotional state.

The DERS-E has been validated in the Chilean context, demonstrating good psychometric properties, with Cronbach’s alpha coefficients ranging from *α* = 0.71 to α = 0.73 (Guzmán-González et al., 2014). For this study, we used a selection of items representing key factors of the scale. The selected items, their underlying factors, and their psychometric properties are described below. This instrument was administered to students aged 13 and older. The selected items from the DERS-E scale, administered to students aged 13 and older, capture five core difficulties in emotion regulation. These include: “*When I feel bad, I feel ashamed of myself for feeling that way”* (Emotional rejection), “*When I feel bad, I lose control over my behavior”* (Emotional dyscontrol), “*When I feel bad, I find it hard to concentrate on other things*” (Emotional interference), “*I pay attention to how I feel*” (Emotional inattentiveness), and “*I have difficulty understanding my feelings*” (Emotional confusion). These items were selected to reflect key emotional regulation challenges experienced by adolescents.

Because selected indicators were used rather than validated short forms, internal consistency coefficients were not estimated for the reduced item sets. Cronbach’s *α* and McDonald’s *ω* are not defined for single-item indicators, and computing α/ω across items representing distinct domains would be psychometrically misleading. Accordingly, all analyses treat these variables as item-level indicators rather than internally consistent scale scores.

### 3.4 Experimental procedure

Procedure. Data were collected in public schools in the municipality of Peñalolén (Santiago, Chile) during October 2024. Prior to data collection, written informed consent was obtained from participants’ legal guardians, and students provided assent. The questionnaires were administered online during regular class hours, and research staff were present throughout the sessions to support the application and answer any questions the students had. Sessions lasted approximately 60 min. The EPOCH Well-being item-level indicators and the VIA Character Strengths item-level indicators were administered to all participants, whereas the items-level indicators derived from CERQ-K was administered to the younger group (8–12 years) and the DERS-E to the older group (13–18 years). Participation was voluntary and anonymous, and no incentives were provided. The study was approved by the Ethics Committee of the School of Psychology, Universidad Adolfo Ibáñez.

### 3.5 Data analysis

We first computed descriptive statistics, including means, standard deviations, and ranges, for all study variables, and inspected distributions and potential outliers. For bivariate associations, we estimated zero-order Pearson correlations between each selected EPOCH wellbeing indicator (Engagement, Perseverance, Optimism, Connectedness, and Happiness) and the selected item-level indicators of emotion regulation derived from the CERQ-K and DERS-E. When distributional assumptions were not met, Spearman’s *ρ* was used as a robustness check. HC3 heteroskedasticity-consistent standard errors were used only in the regression models, not in the bivariate correlation analyses.

To assess the unique statistical contribution of emotion-regulation and character-strength indicators to wellbeing, we fitted five hierarchical linear regression models, one for each selected EPOCH wellbeing indicator. Models were estimated separately for younger students (8–12 years) and older students (13–18 years). This stratification was necessary because different developmentally appropriate emotion-regulation instruments were administered to each age group: selected CERQ-K indicators, reflecting cognitive emotion-regulation strategies, were administered to students aged 8–12 years, whereas selected DERS-E indicators, reflecting emotion-regulation difficulties, were administered to students aged 13–18 years. Because these instruments assess related but distinct aspects of emotion regulation and were administered to different subsamples, separate regression models were used to ensure conceptual and methodological consistency.

In each subsample, Step 1 included covariates: age, gender, and school level. Step 2 added the instrument-specific selected emotion-regulation indicators derived from the CERQ-K or DERS-E, all z-standardized. Step 3 added the 15 selected VIA-based character-strength indicators, also z-standardized and entered simultaneously: Self-knowledge, Perspective, Love of learning, Kindness, Bravery, Enthusiasm, Love, Empathy, Teamwork, Fairness, Prudence, Self-regulation, Hope, Gratitude, and Perseverance. We report standardized regression coefficients (*β*), HC3 robust standard errors, 95% confidence intervals, two-tailed *p* values, and FDR-adjusted *q* values. Multiplicity was controlled using the Benjamini–Hochberg false discovery rate procedure within each outcome-by-stratum model. The incremental contribution of each predictor block was summarized using ΔR^2^: Step 2 ΔR^2^ reflects the variance added by the emotion-regulation indicators beyond covariates, and Step 3 ΔR^2^ reflects the additional variance added by the character-strength indicators beyond covariates and emotion regulation. Full model fit is reported using model-specific *N*, R^2^ at each step, final R^2^, and total ΔR^2^.

Regression assumptions were evaluated through diagnostic checks. We inspected residual plots and standardized residuals to assess linearity, influential observations, and the approximate distribution of residuals. Potential heteroskedasticity was addressed by estimating HC3 robust standard errors in all regression models. Multicollinearity was assessed using variance inflation factors (VIFs) in the final Step-3 models. VIF values were below conventional thresholds in both subsamples, with maximum values of 3.44 in the CERQ-K models and 2.97 in the DERS-E models, suggesting no evidence of severe multicollinearity among predictors.

Missing data were handled using available-case procedures according to the analysis. Descriptive statistics and bivariate correlations were estimated using pairwise available data. Hierarchical regression models were estimated using listwise deletion within each model; therefore, analytic sample sizes vary across models depending on the selected EPOCH indicator, covariates, and item-level predictors included. No imputation was performed.

Given the large sample size, statistical significance was interpreted alongside effect-size magnitude and consistency across models. Correlations were interpreted using conventional benchmarks, with values around |0.10| considered small, around |0.30| moderate, and around |0.50| large. In regression models, emphasis was placed on standardized *β* estimates, 95% confidence intervals, ΔR^2^, and the consistency of effects across domains and subsamples, rather than on *p* values alone. Statistical significance was set at *α* = 0.05 (two-tailed). All analyses were conducted in R (R Core Team, 2020) using RStudio (version 1.3.1073; RStudio Team, 2020).

## 4 Findings

### 4.1 Descriptive results

The total sample comprised 3,569 students (52.1% male, 45.6% female, 2.3% other). The mean age was 12.44 years (*SD* = 3.73; range = 8–18; *n* = 3,569), means (SDs) were: Engagement 2.64 (1.20), Perseverance 2.91 (1.20), Optimism 2.77 (1.25), Connectedness 3.82 (1.25), and Happiness 3.49 (1.27). Among younger group students who completed the selected CERQ-K item-level indicators (1–5; *n* = 2,620), means (SDs) were: Rumination 2.91 (1.41), Guilt 2.81 (1.44), Proactive Thinking 2.93 (1.35), Positive Refocusing 2.55 (1.37), and Perspective Taking 2.66 (1.32). Among older group students who completed the selected DERS-E item-level indicators (1–5; *n* = 1,219), means (SDs) were: Emotional Rejection 2.37 (1.29), Emotional Dyscontrol 2.30 (1.31), Emotional Interference 2.98 (1.35), Emotional Inattention 2.66 (1.24), and Emotional Confusion 2.63 (1.34).

Taken together, the wellbeing indicators showed the highest mean for the selected EPOCH Connectedness indicator (*M* = 3.82, *SD* = 1.25; 1–5 scale). Among younger students, the selected CERQ-K item-level indicators with the highest means were Proactive Thinking (*M* = 2.93, *SD* = 1.35) and Rumination (*M* = 2.91, *SD* = 1.41), whereas Positive Refocusing showed the lowest mean (*M* = 2.55, *SD* = 1.37). Among older students, the selected DERS-E item-level indicator with the highest mean was Emotional Interference (*M* = 2.98, *SD* = 1.35), whereas Emotional Dyscontrol showed the lowest mean (*M* = 2.30, *SD* = 1.31). Sample sizes vary across measures because different emotion-regulation indicators were administered to different subsamples according to age group (CERQ-K-derived indicators in the younger group; DERS-E-derived indicators in the older group).

For the selected VIA-based character-strength indicators (1–5 scale), the highest means were observed for Self-regulation (*M* = 2.71, *SD* = 1.35), Prudence (*M* = 2.69, *SD* = 1.29), and Perspective (*M* = 2.60, *SD* = 1.25), whereas the lowest means were observed for Gratitude (*M* = 2.12, *SD* = 1.20), Perseverance (*M* = 2.21, *SD* = 1.22), and Love (*M* = 2.25, *SD* = 1.25). All items used a 1–5 scale, with higher scores indicating greater endorsement of the corresponding attribute. Next, Table 2 reports two-tailed Pearson correlations between the selected emotion-regulation indicators derived from the CERQ-K and DERS-E and the selected EPOCH wellbeing indicators.

**Table 2:** Pearson correlations between predictors (CERQ-K, DERS-E) and EPOCH domains.

Predictor	Engagement	Perseverance	Optimism	Connectedness	Happiness
Rumination (CERQ-K)	0.19***	0.08***	0.02	0.08***	0.01
Guilt (CERQ-K)	0.16***	−0.00	−0.03	0.08***	−0.06**
Proactive thinking (CERQ-K)	0.09***	0.17***	0.21***	0.14***	0.19***
Positive refocusing (CERQ-K)	0.04*	0.24***	0.24***	0.16***	0.28***
Perspective taking (CERQ-K)	0.09***	0.20***	0.22***	0.12***	0.20***
Emotional rejection (DERS-E)	0.16***	0.03	−0.01	0.04	−0.02
Emotional dyscontrol (DERS-E)	0.11***	0.00	−0.02	0.05	−0.02
Emotional interference (DERS-E)	0.18***	−0.02	−0.05	0.14***	0.02
Emotional inattention (DERS-E)	0.00	0.21***	0.34***	0.23***	0.30***
Emotional confusion (DERS-E)	0.11***	0.02	−0.06	0.08*	−0.02

Pearson’s r with significance indicated by asterisks; two-tailed tests. *p < 0.05, **p < 0.01, ***p < 0.001.

Adaptive cognitive regulation strategies from the selected CERQ-K item-level indicators showed positive, statistically significant associations of small to moderate magnitude with affective and self-regulatory selected EPOCH indicators; for example, Positive Refocusing–Happiness (*r* = 0.28, *p* < 0.001), Positive Refocusing–Optimism (*r* = 0.24, *p* < 0.001), and Perspective Taking–Optimism (*r* = 0.22, *p* < 0.001). A small negative correlation was also observed between Guilt and Happiness (*r* = −0.06, *p* = 0.003). Regarding emotional difficulties on the selected DERS-E item-level indicators, several were positively associated with selected EPOCH item-level indicators, most notably Emotional Inattention with Optimism (*r* = 0.34, *p* < 0.001) and Happiness (*r* = 0.30, *p* < 0.001), as well as with Connectedness (*r* = 0.23, *p* < 0.001) and Perseverance (*r* = 0.21, *p* < 0.001); Emotional Interference was related to Connectedness (*r* = 0.14, *p* < 0.001), and Emotional Rejection to Engagement (*r* = 0.16, *p* < 0.001). Taken together, the pattern suggests that greater use of adaptive cognitive strategies is linked to higher subjective wellbeing, whereas the positive associations between certain emotional difficulties (particularly Emotional Inattention) and selected EPOCH item-level indicators are counterintuitive given the scale’s conceptualization (higher scores = greater difficulty) and may reflect measurement artifacts rather than a substantive effect (see Discussion and Limitations).

### 4.2 Hierarchical regressions

We estimated five hierarchical linear regressions, one for each selected EPOCH wellbeing indicator, separately in the CERQ-K and DERS-E subsamples. Models were stratified by instrument because the selected CERQ-K indicators were administered to the younger group (8–12 years), whereas the selected DERS-E indicators were administered to the older group (13–18 years). In each subsample, Step 1 included covariates: age, gender, and school level. Step 2 added the instrument-specific selected emotion-regulation indicators entered simultaneously. For the CERQ-K subsample, these were Rumination, Guilt, Proactive Thinking, Positive Refocusing, and Perspective Taking. For the DERS-E subsample, these were Emotional Rejection, Emotional Dyscontrol, Emotional Interference, Emotional Inattention, and Emotional Confusion. Step 3 added the 15 selected VIA-based character-strength indicators entered simultaneously after z-standardization.

As shown in Table 3, the Step-1 covariate models explained between 1 and 5% of the variance across the selected EPOCH wellbeing indicators. Adding the selected emotion-regulation indicators in Step 2 increased explained variance in both subsamples, with ΔR^2^ values ranging from 0.039 to 0.083 in the CERQ-K models and from 0.042 to 0.104 in the DERS-E models. Adding the selected VIA-based character-strength indicators in Step 3 produced further increments in explained variance, particularly for Perseverance, Happiness, and Optimism. Final R^2^ values for the full Step-3 models ranged from 0.080 to 0.192 in the CERQ-K subsample and from 0.074 to 0.223 in the DERS-E subsample.

**Table 3:** Hierarchical model fit and incremental explained variance including emotion-regulation and character-strength indicators.

Stratum	EPOCH indicator	*N*	R^2^ step 1	R^2^ step 2	ΔR^2^ step 2	R^2^ step 3/final R^2^	ΔR^2^ step 3	ΔR^2^ total
CERQ-K/younger group	Engagement	2,498	0.019	0.065	0.046	0.080	0.015	0.061
CERQ-K/younger group	Perseverance	2,498	0.045	0.113	0.068	0.174	0.062	0.130
CERQ-K/younger group	Optimism	2,498	0.053	0.127	0.074	0.160	0.033	0.107
CERQ-K/younger group	Connectedness	2,498	0.011	0.049	0.039	0.082	0.033	0.071
CERQ-K/younger group	Happiness	2,498	0.043	0.126	0.083	0.192	0.066	0.149
DERS-E/older group	Engagement	1,044	0.015	0.058	0.042	0.074	0.017	0.059
DERS-E/older group	Perseverance	1,044	0.018	0.069	0.051	0.163	0.093	0.144
DERS-E/older group	Optimism	1,044	0.041	0.145	0.104	0.223	0.078	0.182
DERS-E/older group	Connectedness	1,044	0.009	0.079	0.070	0.139	0.060	0.130
DERS-E/older group	Happiness	1,044	0.043	0.125	0.082	0.176	0.051	0.133

Step 1 included covariates: age, gender, and school level/grade. Step 2 added the instrument-specific selected emotion-regulation indicators derived from the CERQ-K or DERS-E. Step 3 added the 15 selected VIA-based character-strength indicators entered simultaneously. ΔR^2^ Step 2 = R^2^ (Step 2) − R^2^ (Step 1). ΔR^2^ Step 3 = R^2^ (Step 3) − R^2^ (Step 2). ΔR^2^ Total = R^2^ (Step 3) − R^2^ (Step 1). R^2^ Step 3 corresponds to the final full model. Predictors were z-standardized, and HC3 robust standard errors were used. Because CERQ-K and DERS-E models were estimated in distinct subsamples, cross-stratum comparisons should be interpreted descriptively only.

Given the large sample size, we interpret these findings primarily in terms of effect-size magnitude and consistency across models rather than statistical significance alone. Overall, the largest final model fit was observed for Happiness in the CERQ-K subsample and for Optimism in the DERS-E subsample. Because CERQ-K and DERS-E models were estimated in distinct subsamples, cross-stratum comparisons should be interpreted descriptively only.

As shown in the final Step-3 models, selected VIA-based character-strength indicators added explanatory value beyond covariates and emotion-regulation indicators. The specific character-strength indicators that remained significant after FDR correction are summarized in Table 4.

**Table 4:** FDR-significant character-strength indicators in the final step-3 models.

EPOCH domain	Strength	β	95% CI	SE	*p*	*q*(FDR)	*n*
Perseverance	Love of learning	0.149	[0.100, 0.198]	0.025	<0.001	<0.001	2.498
Perseverance	Perseverance	0.083	[0.033, 0.133]	0.026	0.001	0.007	2.498
Optimism	Hope	0.123	[0.069, 0.177]	0.027	<0.001	<0.001	2.498
Connectedness	Love	0.118	[0.065, 0.171]	0.027	<0.001	<0.001	2.498
Happiness	Enthusiasm	0.147	[0.094, 0.199]	0.027	<0.001	<0.001	2.498
Happiness	Love	0.104	[0.054, 0.155]	0.026	<0.001	<0.001	2.498
Happiness	Teamwork	0.082	[0.033, 0.132]	0.025	0.004	0.004	2.498
Panel B: DERS-E older group							
Perseverance	Love of learning	0.236	[0.152, 0.320]	0.043	<0.001	<0.001	1.044
Optimism	Hope	0.186	[0.093, 0.279]	0.048	<0.001	0.001	1.044
Optimism	Self-knowledge	0.121	[0.044, 0.198]	0.039	0.002	0.16	1.044
Connectedness	Teamwork	0.122	[0.042, 0.203]	0.041	0.003	0.32	1.044
Happiness	Teamwork	0.108	[0.032, 0.184]	0.039	0.005	0.42	1.044

“Panel A: CERQ-K younger group” above the first set of rows in Table 4 (the rows with *n* = 2,498), parallel to the existing “Panel B: DERS-E older group” heading. Coefficients are standardized β estimates from the final Step-3 models. Panel A reports models estimated in the younger group, in which the selected CERQ-K emotion-regulation indicators were included in Step 2 before adding character-strength indicators in Step 3. Panel B reports models estimated in the older group, in which the selected DERS-E emotion-regulation indicators were included in Step 2 before adding character-strength indicators in Step 3. All models included age, gender, and school level as covariates. HC3 robust standard errors were used. Confidence intervals are 95% CIs for β. FDR-adjusted q values were computed using the Benjamini–Hochberg correction within each outcome-by-stratum model. Only character-strength indicators with *q* < 0.05 are displayed.

## 5 Discussion

The present study sought to examine the intricate relationships between selected item-level indicators of emotional regulation, character strengths, and wellbeing in children and adolescents attending public primary and secondary schools in the municipality of Peñalolén, Chile. Rather than administering the complete versions of the original instruments, the study used selected indicators derived from the EPOCH framework to represent multidimensional wellbeing, from the CERQ-K to represent cognitive emotion-regulation strategies in children, from the DERS-E to represent emotion-regulation difficulties in adolescents, and from a VIA-based measure to represent character strengths. This item-level indicator approach aimed to provide preliminary empirical evidence from a Latin American context on how observed indicators of psychological resources and wellbeing are associated in school-aged populations. To our knowledge, this is the first study in Latin America to examine these selected wellbeing, emotion-regulation, and character-strength indicators jointly in children and adolescents.

### 5.1 General overview of findings

Descriptive results indicated that students’ wellbeing levels fell within moderate ranges across all EPOCH wellbeing indicators, with *Connectedness* and *Happiness* emerging as the highest domains and *Engagement* and *Optimism* as the lowest. This pattern is consistent with school and -based studies showing that relational aspects of wellbeing (e.g., belonging, positive relationships) are often more robust than agentic or future-oriented components among adolescents in collectivist or community-oriented contexts (Waters and Loton, 2019). Within emotional regulation, younger students exhibited a moderate-to-low use of adaptive cognitive strategies, particularly positive refocusing and perspective taking, whereas older students reported low-to-moderate emotional difficulties, with emotional interference as their main challenge. These age-related differences suggest that as students mature, their emotional awareness increases, but so does the intensity and complexity of emotional experience, requiring more advanced regulation skills (Gross, 2015; Silvers, 2022).

At the correlational level, higher use of adaptive cognitive emotion-regulation strategies was associated with higher scores on selected EPOCH wellbeing indicators, replicating international evidence that cognitive reappraisal, planning, and acceptance are robust predictors of adolescent wellbeing and psychological adjustment in youth (Martínez-Líbano et al., 2025). Conversely, greater emotional difficulties, particularly interference and lack of clarity, were linked to lower wellbeing, consistent with evidence that limited access to emotion-regulation strategies is a central feature of emotion-dysregulation networks and that difficulties in emotion regulation are associated with depressive symptoms in adolescents (Ruan et al., 2023; Kökönyei et al., 2024).

A counterintuitive association emerged for inattention to emotions, which showed a small positive correlation with wellbeing. We interpret this finding cautiously, as several methodological factors are likely to account for it. First, this DERS-E subscale was represented by a single, reverse-keyed item (“I pay attention to how I feel”), which limits its construct validity and introduces ambiguity in scoring direction; a single item cannot reliably capture the lack-of-emotional-awareness construct as defined in the full scale. Second, the abbreviated item set may have altered the psychometric properties of the original instrument (see Limitations), and response biases such as acquiescence or social desirability cannot be ruled out. We therefore refrain from substantive interpretations of this association. Although one might speculate that reduced self-focused attention could relate to lower rumination (Mor and Winquist, 2002), our design included no measures of mindfulness, meta-awareness, or adaptive detachment, and the data do not support such an interpretation. This isolated association should be regarded as preliminary and verified with the complete DERS-E in future research.

### 5.2 Developmental distinctions in predictive patterns

Regression analyses revealed developmental differences consistent with lifespan models of emotion regulation. Among younger students, adaptive strategies such as positive refocusing and taking perspective predicted higher *Happiness, Optimism,* and *Perseverance*, indicating that cognitive reframing and flexible perspective-taking may serve as early foundations for optimism and sustained effort.

In contrast, for older students, emotional difficulties (especially interference and lack of clarity) became stronger predictors of wellbeing, suggesting that during adolescence the *absence* of dysregulation becomes more critical than the *presence* of adaptive strategies. This developmental shift aligns with neurocognitive research showing that adolescence is characterized by heightened affective reactivity and still-maturing prefrontal regulatory systems (Silvers, 2022). Interventions for older students may therefore require greater focus on managing interference and emotional overwhelm rather than solely fostering cognitive reframing.

### 5.3 The incremental value of character strengths

Adding the selected VIA-based character-strength indicators after the covariates and the selected emotion-regulation indicators derived from the CERQ-K and DERS-E accounted for additional variance in the selected EPOCH wellbeing indicators. This incremental contribution was particularly evident for Perseverance, Optimism, and Happiness. The pattern was more pronounced in the older group, especially for Perseverance and Optimism, where DERS-E-derived emotion-regulation difficulty indicators showed stronger statistical associations with wellbeing indicators. This suggests that, in our data, strengths were not merely associated with wellbeing as passive buffers but showed independent positive associations with indicators of flourishing (Niemiec, 2023). Descriptively, emotion-regulation variables and strengths accounted for distinct portions of variance in wellbeing: regulation variables related to how emotions are managed, whereas strengths related to motivational and dispositional resources. We interpret this complementary pattern tentatively; a formal two-pathway account would require confirmatory modeling beyond the present cross-sectional data.

### 5.4 Which strengths truly matter?

Table 4 provides the most nuanced insight of this research. By entering all strengths simultaneously and controlling for age, gender, and emotional-regulation variables (selected DERS-E, CERQ-K item-level indicators), it isolates the *unique* contribution of each strength to wellbeing, revealing which qualities remained uniquely associated with flourishing indicators when all others were held constant. Across both age groups, a consistent constellation of strengths emerged: Love of Learning, Hope, Self-Regulation, Enthusiasm, Love, and Teamwork.

1. Love of Learning → Perseverance (younger and older groups).

This relationship underscores the cognitive-motivational roots of perseverance. Students who find intrinsic pleasure in acquiring knowledge are more likely to display sustained effort and commitment. Love of Learning reflects curiosity, mastery orientation, and autonomy, core components of self-determined motivation (Deci and Ryan, 2000). Its predictive value for Perseverance in both age groups suggests that cultivating curiosity early can translate into long-term engagement and grit. Educational practices that nurture inquiry, reflection, and autonomy may thus indirectly foster academic and psychological resilience.

2. Hope → Optimism (+ self-regulation in the older group).

Hope encompasses goal-directed thinking, agency, and perceived pathways to achieve desired outcomes (Snyder, 2002). Its robust link with Optimism confirms that hopeful students not only expect positive outcomes but also believe in their capacity to generate them. In secondary school, Self-Regulation joined Hope as a significant predictor for the dimension of optimism, suggesting that managing impulses and emotions may be relevant for maintaining optimism over time. This pattern is consistent with the idea of mutually reinforcing processes, in which hope relates to motivation and regulation to persistence when obstacles arise. Together they embody the adaptive capacity to anticipate the future with confidence and discipline.

3. Enthusiasm, love, and teamwork → Connectedness and happiness.

These socio-affective strengths underscore the relational dimensions of wellbeing. Enthusiasm, conceptually akin to Zest, captures vitality and joyful engagement with life; Love embodies emotional warmth and closeness; and Teamwork reflects collaboration and collective efficacy. Their joint predictive power for Connectedness and Happiness suggests that students’ wellbeing is anchored in belonging and shared purpose. In Chilean public-school contexts, often characterized by strong community ties, these interpersonal strengths may be linked to indicators of collective flourishing.

The simultaneous inclusion of these strengths is consistent with a distinction between two groups of wellbeing resources. Cognitive–transcendence strengths, such as *Love of Learning, Hope,* and *Self-Regulation*, were associated with meaning-making, self-determination, and goal pursuit, supporting students’ sense of purpose and perseverance. In contrast, affective–interpersonal strengths, including *Enthusiasm, Love,* and *Teamwork* were associated with nurture emotional vitality, belonging, and positive relationships. This distinction mirrors the configuration of the EPOCH model: cognitive–transcendence strengths align more closely with the dimensions of *Perseverance* and *Optimism*, whereas affective–interpersonal strengths correspond to *Connectedness* and *Happiness*. Together, these clusters suggest that flourishing reflects on the interplay between intrapersonal mastery and interpersonal connection.

### 5.5 Alignment with global evidence

The prominence of Hope and Enthusiasm (Zest) resonates strongly with the meta-analytic evidence presented by Casali and Feraco (2025), who aggregated over 100 studies examining the associations between VIA strengths, wellbeing, and mental health. Their results showed that nearly all strengths correlate positively with wellbeing, but Hope (r ≈ 0.52) and Zest (r ≈ 0.52) stood out as the most influential. Importantly, correlations with wellbeing were substantially stronger than those with the absence of psychopathology, underscoring that strengths represent distinct pathways to positive functioning rather than mere opposites of distress.

The current findings extend this global evidence to a Latin-American educational context, identifying Hope (predicting Optimism) and Enthusiasm/Zest (predicting Happiness and Connectedness) as core mechanisms of youth flourishing. Additionally, Love of Learning and Teamwork broaden the picture by linking cognitive engagement and social collaboration with perseverance and relational satisfaction. Together, these results suggest a culturally resilient structure of strengths: although contextual expression may vary, the broad pattern of strength–wellbeing associations observed here is consistent with meta-analytic evidence from other contexts (Casali and Feraco, 2025).

### 5.6 Theoretical implications

The observed patterns can be interpreted through multiple theoretical lenses. First, the *broaden-and-build* theory (Fredrickson, 2001) provides an explanatory framework for how positive emotions generated by strengths amplify cognitive and social resources. For instance, enthusiasm and love elicit approach motivation, broaden attention, and facilitate the building of supportive relationships that, in turn, reinforce happiness and connectedness.

Second, our findings can be read in terms of a broad distinction between regulatory processes that manage affective experience (here, the selected CERQ-K and DERS-E indicators) and motivational or dispositional resources that provide purpose and direction (here, the selected VIA-based character-strength indicators). We advance this as an interpretive lens rather than as a formal model and note that the present cross-sectional data cannot establish the reciprocal dynamics it implies.

Importantly, to our knowledge, few studies have empirically examined which specific character strengths best predict the dimensions of the EPOCH model while simultaneously accounting for emotion-regulation capacities. This cross-domain integration constitutes one of the novel contributions of the present research. The findings indicate that maintaining a focus on strengths not only improves wellbeing outcomes but may also create psychological conditions for developing more adaptive regulatory strategies. Certain strengths, such as *Self-Regulation, Hope,* and *Love of Learning*, are theoretically linked to effective emotional management, suggesting that they could serve as gateways to broader self-regulatory competence. Future studies should therefore explore whether particular strengths directly foster better regulation skills or moderate the impact of emotional difficulties on wellbeing. Understanding these reciprocal pathways may clarify how strength-based interventions can operate simultaneously as mechanisms for emotional growth and as catalysts for sustained flourishing in adolescence.

Third, situating these findings in relation to broader models of flourishing allows for a clearer understanding of their conceptual significance. While Seligman’s (2011) PERMA framework was originally developed to capture flourishing in adults, the EPOCH model provides a developmentally appropriate formulation for adolescents, encompassing Engagement, Perseverance, Optimism, Connectedness, and Happiness (Kern et al., 2015; Kern et al., 2016). The present results reveal how specific character strengths map onto these domains, reinforcing the structural coherence of positive-psychology models across age groups. *Hope* and *Self-Regulation* are closely tied to Optimism and Perseverance, reflecting an agentic orientation toward goals and adaptive coping; *Love of Learning* aligns with Engagement and Perseverance, emphasizing curiosity and mastery motivation; and interpersonal strengths such as *Love, Teamwork,* and *Enthusiasm* correspond to Connectedness and Happiness, reflecting the relational and emotional dimensions of flourishing.

This convergence suggests that the pathways to wellbeing identified in adult populations, meaning, accomplishment, engagement, positive emotion, and relationships are mirrored developmentally within the adolescent EPOCH structure. In this sense, EPOCH can be viewed not as a distinct model but as a contextualized translation of PERMA that captures the specific expressions of flourishing during adolescence, when social belonging, optimism, and perseverance become particularly salient for psychological growth.

### 5.7 Contextual and cultural considerations

Conducted within the Chilean public-school system, this study adds valuable regional evidence to a field dominated by research from North America and Europe. Latin-American educational environments often emphasize collectivist values, solidarity, and community interdependence. The prominence of relational strengths (Love, Teamwork, Enthusiasm) in predicting Connectedness and Happiness may therefore reflect cultural emphases on interpersonal harmony and communal success over individual achievement. Similar patterns have been reported in Peruvian and Mexican samples in Adler’s (2016) cross-cultural work on wellbeing curricula, where relational and civic strengths yielded the greatest gains in school satisfaction.

At the same time, the predictive value of cognitive-motivational strengths such as Love of Learning and Hope suggests that fostering autonomy and mastery remains crucial, even in collectivist contexts. The interplay between agency and communion between striving and belonging may thus represent the hallmark of flourishing in Latin-American youth.

### 5.8 Educational and applied implications

The results yield several actionable insights for policy and practice.

1. Promoting emotional regulation across childhood and adolescence.

Promoting emotional regulation throughout the school years is fundamental for fostering psychological wellbeing and preventing mental health and relational difficulties. Research consistently shows that early emotional-regulation training predicts greater social competence and a lower risk of internalizing and externalizing problems later in life (Durlak et al., 2011; Fomina et al., 2020). For the younger group, programs should incorporate explicit training in adaptive strategies such as perspective taking, and positive refocusing. Embedding these practices into everyday classroom routines helps children develop emotional awareness, optimism, and perseverance while reducing frustration and disengagement. As students transition into adolescence, the focus should expand to cultivating *hope*, and *self-regulation*, which were among the strongest predictors of *optimism* in this study. Taken together, these strategies highlight the importance of embedding emotional-regulation education within the broader school curriculum not only to promote wellbeing, but also to prevent mental health problems and improve school coexistence, contributing to safer, and resilient learning environments.

2. The importance of promoting character strengths for children’s wellbeing.

Promoting character strengths in schools is essential for nurturing children’s overall wellbeing and healthy socioemotional development. Initiatives that intentionally cultivate cooperation, empathy, and collective projects help strengthen Teamwork, Love, and Enthusiasm, strengths that foster belonging, security, and mutual care, all of which are fundamental psychological needs in childhood (Park and Peterson, 2006). Developing these relational and communal strengths supports positive peer relationships, improves classroom climate, and enhances emotional resilience, thereby protecting children against stress, loneliness, and social exclusion (Waters and Loton, 2019). Moreover, as Niemiec (2023) notes, character strengths not only buffer difficulties but actively promote flourishing by connecting young people to values, meaning, and constructive engagement with others. In the context of Chile’s public education system where community and social identity are central, strengths-based approaches can therefore contribute both to individual wellbeing and to the collective flourishing of school communities. Integrating the systematic promotion of character strengths from early educational stages provides children with enduring personal and interpersonal resources that support positive mental health and wellbeing, serving as a proactive strategy for the prevention of emotional difficulties and the promotion of wellbeing.

3. Integrative wellbeing curricula.

The convergence of emotional regulation and character strengths advocates for a comprehensive approach to positive education. Interventions should move beyond isolated workshops to a systemic and sustained framework that involves all members of the educational community, including teachers, families, school leaders, and support staff working collaboratively to promote student and collective wellbeing (Waters and Loton, 2019). Evidence from a recent review by Vella-Brodrick et al. (2025) indicates that long-term programs those lasting one academic year or more produce stronger and more enduring effects on students’ eudaimonic wellbeing and psychological health than short-term or one-off interventions, which tend to yield smaller and less sustainable gains. These findings support the development of whole-school, long-term strategies that continuously measure and strengthen both emotional-regulation skills and character strengths as the most effective pathway toward lasting improvements in student wellbeing.

4. Measurement and evaluation.

Given that strengths and emotion regulation explain complementary portions of variance in wellbeing, future wellbeing assessments in schools should include both constructs to provide a complete picture of students’ socioemotional health. The use of validated instruments such as EPOCH, CERQ-K, and DERS-E can support longitudinal monitoring and evidence-based decision-making.

### 5.9 Limitations and directions for future research

Several limitations must be acknowledged. First, the cross-sectional design precludes causal inference; longitudinal and experimental designs are needed to determine whether improvements in strengths and emotion regulation lead to sustained increases in wellbeing. Second, reliance on self-report measures introduces potential biases of social desirability and common method variance. Future studies should incorporate teacher ratings, peer reports, or behavioral and physiological indicators of regulation. Third, while the sample encompassed multiple schools within one municipality, broader replication across diverse Chilean regions and other Latin-American countries would strengthen generalizability.

Analytically, entering all 24 strengths simultaneously may obscure potential mediational or synergistic relationships. Future research could employ structural-equation modeling to test whether strengths mediate or moderate the effects of emotional regulation on wellbeing. For example, *Hope* may mediate the pathway from adaptive regulation to Optimism, whereas *Teamwork* might buffer the negative effects of emotional interference on Connectedness. Additionally, longitudinal designs could explore developmental sequencing whether strengths precede improvements in regulation or vice versa.

Use of selected items and reliability estimation: A major limitation of this study is the use of selected items rather than the complete validated instruments. This approach was necessary because schools provided only a limited time window for data collection during regular class hours, and the survey had to remain brief enough to be feasible across 15 municipal schools and different age groups. Item selection was guided by theoretical relevance and prior psychometric evidence, including factor loadings from validation studies when available. Nevertheless, the selected indicators should not be interpreted as validated short forms. Single-item indicators cannot fully capture the breadth, reliability, or latent structure of the original EPOCH, CERQ-K, DERS-E, or VIA-based measures. Consequently, the findings should be interpreted as associations among selected indicators of wellbeing, emotion regulation, and character strengths, rather than as evidence about the complete latent constructs. Internal consistency coefficients such as Cronbach’s *α* or McDonald’s *ω* could not be computed for constructs represented by single items. Moreover, because the selected items within each instrument represented conceptually distinct domains rather than a unidimensional construct, reporting a single α/ω for each reduced item set would have been psychometrically misleading. Future studies should replicate these findings using the full validated instruments or formally developed short forms, allowing estimation of reliability, dimensionality, latent structure, and measurement invariance across age groups.

Finally, the heterogeneity of effect sizes reported by Casali and Feraco (2025) underscores the need to examine cultural and contextual moderators such as socioeconomic status, school climate, and family functioning. Integrating qualitative methods could enrich understanding of how students experience and express their strengths within their sociocultural environments.

## 6 Conclusion

This study advances the empirical understanding of child and adolescent wellbeing by showing that emotional regulation and character strengths are jointly associated with flourishing within the Chilean public-education context. Among younger students, the use of adaptive cognitive strategies was associated with higher wellbeing, whereas among adolescents it was the relative absence of emotion-regulation difficulties, rather than the presence of adaptive strategies, that was most consistently associated with wellbeing. When personal strengths were considered, their contribution was not marginal: *Love of Learning* and *Hope* were associated with perseverance and optimism; *Self-Regulation* with goal-related dimensions; and *Enthusiasm, Love,* and *Teamwork* with happiness and connectedness.

Together, these cognitive, motivational, and relational components were associated with the multiple dimensions of flourishing assessed in this study. In light of global evidence, especially the Casali and Feraco (2025) meta-analysis identifying Hope and Zest as universal pillars of wellbeing, the present findings highlight the importance of contextualizing strength-based interventions within Latin-American schools while maintaining alignment with international frameworks such as PERMA. Moving forward, educational systems should embrace an integrative vision of mental health one that treats emotion regulation and character development not as peripheral programs but as central missions of schooling. By doing so, schools can become ecosystems that cultivate hope, curiosity, vitality, and belonging, equipping children and adolescents not only to cope with challenges but to live meaningful, connected, and joyful lives.

## Data Availability

The original contributions presented in the study are included in the article/supplementary material, further inquiries can be directed to the corresponding author.
